# Low‐Intensity Pulsed Ultrasound Alleviation of LPS‐Induced Depression‐Like Behavior via Microglial P2X4R Inhibition and BDNF/TrkB Pathway Activation

**DOI:** 10.1002/cns.70786

**Published:** 2026-02-11

**Authors:** Yuanli Wang, Xinyue Meng, Xinyi Zhang, Ruilin Chen, Mianzhi Zhang, Deli Yang, Wanke Zou, Paul K. Chu, Chengbiao Ding, Hongrui Zhan

**Affiliations:** ^1^ Department of Rehabilitation Medicine The Fifth Affiliated Hospital of Sun Yat‐Sen University Zhuhai City Guangdong Province China; ^2^ Department of Rehabilitation Medicine The Second Hospital of Anhui Medical University Hefei Anhui China; ^3^ Anhui Medical University Hefei Anhui China; ^4^ Department of Rehabilitation Medicine The Fifth People's Hospital of Zhuhai Zhuhai City Guangdong Province China; ^5^ Department of Physics, Plasma Laboratory City University of Hong Kong Kowloon, Hong Kong China; ^6^ Department of Biomedical Engineering City University of Hong Kong Kowloon, Hong Kong China; ^7^ Department of Materials Science and Engineering City University of Hong Kong Kowloon, Hong Kong China

**Keywords:** BDNF/TrkB, depression, low‐intensity pulsed ultrasound, neuroinflammation, P2X4R

## Abstract

**Objective:**

Low‐intensity pulsed ultrasound (LIPUS) shows promising anti‐inflammatory and neuroprotective effects for different types of neurological disorders. This study aims to investigate the therapeutic effects of LIPUS on LPS‐induced depression‐like behavior and neuroinflammation and to elucidate the underlying molecular mechanisms.

**Methods:**

A depressive mouse model is established by intraperitoneal injection of LPS (1.0 mg/kg/day for 7 days). LIPUS is applied to the hippocampal region (30 min/day). Behavioral assessments include the open field test (OFT), forced swim test (FST), and tail suspension test (TST). Molecular analyses, including Western blotting, immunofluorescence, and qPCR, are performed to evaluate the expression of P2X4R, IBA1, inflammatory cytokines (IL‐1β, IL‐6, TNF‐α), BDNF/TrkB signaling pathway, and apoptosis‐related proteins (Bax, Bcl‐2). The involvement of P2X4R is further examined using ivermectin (IVM), a selective P2X4R agonist.

**Results:**

LIPUS significantly alleviates the LPS‐induced depression‐like behavior, suppresses hippocampal pro‐inflammatory cytokine expression, inhibits microglial activation, and reduces neuronal apoptosis. Mechanistically, LIPUS downregulates P2X4R and IBA1, upregulates BDNF protein levels and TrkB phosphorylation, and modulates the Bax and Bcl‐2 expression. Co‐localization studies confirm that P2X4R is predominantly expressed in microglia, and LIPUS markedly reduces the overlap. Notably, the anti‐inflammatory, neuroprotective, and antidepressant effects of LIPUS are significantly attenuated by IVM, highlighting the critical role of P2X4R suppression in mediating therapeutic effects.

**Conclusion:**

LIPUS mitigates LPS‐induced neuroinflammation, neuronal apoptosis, and depression‐like behavior by targeting microglial P2X4R and activating the BDNF/TrkB pathway. The findings provide mechanistic insights and demonstrate that LIPUS is a promising non‐pharmacological intervention for depression, underscoring the translational potential of P2X4R as a therapeutic target.

## Graphical abstract

LIPUS alleviates LPS‐induced depressive‐like behavior by suppressing microglial P2X4R in the hippocampus. P2X4R downregulation restores BDNF/TrkB signaling, thereby reducing microglial activation, pro‐inflammatory cytokine release, and neuronal apoptosis.

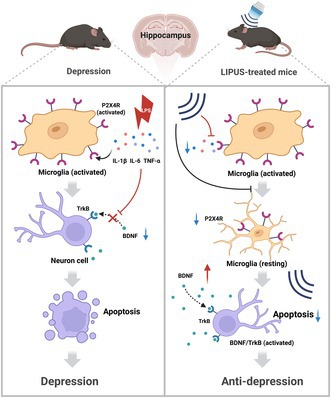

## 1 Introduction

Depression is a complex, prevalent, and debilitating psychiatric disorder that adversely affects the daily lives of millions of people worldwide and significantly diminishes their quality of life. By 2030, it is projected to become one of the top three contributors to the global burden of diseases [1], and it remains a leading cause of disability and suicide [2]. Characterized by persistent low mood, hopelessness, anhedonia, and diminished motivation, depression currently afflicts an estimated 350 million individuals globally [3, 4]. Alarmingly, approximately one‐third of patients fail to respond to conventional antidepressant therapies [5, 6]. Growing evidence suggests that non‐pharmacological neuromodulatory techniques may modulate dysfunctional brain connectivity [7, 8], consequently offering promising alternatives for therapeutic intervention in depression.

The pathogenesis of depression involves an initial stress response followed by a cascade of biological alterations [9], among which neuroinflammation has recently gained significant attention as a secondary pathological process. There is growing evidence that neuroinflammation plays a pivotal role in the onset and progression of depression, potentially through mechanisms involving the release of pro‐inflammatory cytokines [10, 11], activation of microglia within the brain [12], and disruption of neural circuit function. Notably, patients with depression often exhibit structural and functional abnormalities in brain regions such as the prefrontal cortex and hippocampus [13], which are also areas of heightened neuroinflammatory activity. P2X4 receptors (P2X4R), a critical member of the P2X purinergic receptor family, primarily mediate sodium and calcium influx and potassium efflux. Recent studies have identified P2X4R as a key regulator in the pathophysiology of depression [14] and associated neuroinflammatory processes [15, 16], with markedly elevated expression particularly observed in activated microglia [17, 18]. Through P2X4R channels, microglia detect extracellular ATP signals, trigger inflammatory cascades, and release pro‐inflammatory cytokines [19], contributing to neuronal dysfunction and depression‐like behavior. The pharmacological blockade of P2X4R inhibits excessive microglial activation, enhances brain‐derived neurotrophic factor (BDNF) secretion, attenuates neuroinflammation, and alleviates depressive symptoms [20]. Therefore, P2X4R represents a promising therapeutic target in the treatment of depression.

Low‐intensity pulsed ultrasound (LIPUS), a non‐invasive physical intervention, has garnered increasing attention recently for its potential applications in neuropsychiatric disorders [21, 22]. Previous studies have demonstrated that LIPUS can penetrate the skull and precisely target specific brain regions to modulate neurons and glial cells [23]. There is evidence of the efficacy of LIPUS in alleviating neuroinflammation, modulating neurotrophic factors, enhancing synaptic plasticity, and exerting antidepressant‐like effects [24, 25]. Clinical studies have demonstrated the therapeutic efficacy of LIPUS in Parkinson's disease and other disorders [26, 27]. However, the underlying mechanism by which LIPUS ameliorates neuroinflammation in depression remains unclear. Based on previous studies, we hypothesize that LIPUS may alleviate neuroinflammation in depression by modulating P2X4 receptor expression and its downstream signaling pathways.

This study hypothesizes that hippocampal‐targeted LIPUS can modulate P2X4R expression, thereby influencing neuroinflammatory responses and apoptosis in microglia. Specifically, we propose that LIPUS downregulates P2X4R expression in activated microglia and, through the activation of the CaMKII/CREB pathway, subsequently enhances BDNF/TrkB signaling. This modulation ultimately reduces neuroinflammation and apoptosis, suggesting that LIPUS may serve as a potential therapeutic intervention for neuroinflammation associated with depression.

## 2 Materials and Methods

### 2.1 Animals

Adult male C57BL/6J mice (8–10 weeks, 20–25 g) were obtained from Qingyuan Biotechnology Co. Ltd. (Hangzhou, China). The mice were housed under specific pathogen‐free (SPF) conditions with ad libitum access to food and water and maintained on a 12/12 h light/dark cycle. All the experimental procedures followed the Guide for the Care and Use of Laboratory Animals published by the U.S. National Institutes of Health.

### 2.2 Drug Administration and Ultrasound Intervention

The experiment is divided into two parts. The first part consists of three groups: Control group, LPS group, and LPS + LIPUS group (hereafter referred to as the LIPUS group). The second part includes LPS group, LIPUS group, LPS + LIPUS + IVM group (hereafter referred to as the IVM group), and LPS + LIPUS + KN‐93 group (hereafter referred to as the KN‐93 group). Each group contains 8 samples (*n* = 8). To simulate the neuroinflammatory pathophysiology associated with depression, a lipopolysaccharide (LPS)‐induced depressive mouse model was established based on previously reported protocols [28]. LPS (GC205009‐10 mg, Servicebio) was dissolved in sterile saline and administered intraperitoneally at 1.0 mg/kg/day for 7 days. Ivermectin (IVM, HY‐15310, MCE), a P2X4R agonist, was diluted in 0.5% DMSO to a final concentration of 1.25 mg/mL [17]. IVMwas administered intraperitoneally at 1.0 mg/kg/day, 30 min after each LPS injection, for 7 days. KN‐93 (HY‐15465, MCE), a CaMKII inhibitor, was diluted in PBS to a concentration of 2.5 mg/mL and administered at a dose of 1 mg/kg/day via intraperitoneal injection, starting 30 min after LPS administration, for a total of 7 days. LIPUS stimulation was delivered using an ultrasound transducer operating at 800 kHz, coupled with a custom‐fabricated polymethyl methacrylate (PMMA) collimator, which was fixed over the targeted cranial region of the mouse. The diameter of the collimator tip was 3 mm. During ultrasound application, the transducer was connected to the collimator, which was filled with degassed, deionized water to facilitate efficient acoustic energy transmission to the brain. Based on previously published studies [25, 29], the ultrasound parameters were set as follows: pulse repetition frequency of 100 Hz, duty cycle of 10%, and total stimulation duration of 30 min per session.

### 2.3 Western Blot Analysis

The mice were anesthetized and perfused transcardially with 1 mol/L phosphate‐buffered saline (PBS). The brains were rapidly dissected, and hippocampal tissues were isolated. The tissues were homogenized in RIPA lysis buffer (Beyotime, Shanghai, China) and centrifuged at 12,000 ***g*** for 15 min at 4°C. The protein concentrations were determined using a BCA Protein Assay Kit (Thermo Fisher Scientific, Waltham, MA, USA). Western blotting was performed according to the manufacturer's protocol.

Briefly, equal amounts of protein (40 μg) from each sample were mixed with the loading buffer, separated by SDS‐PAGE, and transferred onto the nitrocellulose membranes. The membranes were blocked with 5% non‐fat milk at room temperature for 1 h and incubated overnight at 4°C with the following primary antibodies: TNF‐α (1:1000; AF7014; Affinity), IL‐6 (1:1000; DF6087; Affinity), IL‐1β (1:1000; AF4006; Affinity), Bax (1:1000; AF0120; Affinity), Bcl‐2 (1:1000; AF6139; Affinity), P2X4R (1:1000; 13,534–1‐AP; Proteintech), IBA1 (1:1000; DF6442; Affinity), BDNF (1:1000; DF6387; Affinity), phospho‐TrkB (p‐TrkB, 1:1000; AF3461; Affinity), TrkB (1:1000; AF6461; Affinity), phospho‐CaMKII (p‐CaMKII, 1:1000; AF3493; Affinity), CaMKII (1:1000; AF6493; Affinity), phospho‐CREB (p‐CREB, 1:1000; AF3189; Affinity), CREB (1:1000; AF6188; Affinity), and β‐actin (1:5000; AF7018; Affinity). The membranes were then incubated with horseradish peroxidase (HRP)‐conjugated secondary antibodies (Beyotime, Shanghai, China) at room temperature for 1 h. The protein bands were visualized using an ECL Plus chemiluminescence detection kit (Affinity, KF8003), and band intensity was quantified using ImageJ software (NIH, Bethesda, MD, USA).

### 2.4 Immunofluorescence Staining

The mice were anesthetized and perfused transcardially with 20 mL of ice‐cold 0.1 mol/L PBS, followed by 4% paraformaldehyde (PFA). The brain tissues were collected, post‐fixed in 4% PFA overnight, and cryoprotected in 30% sucrose at 4°C for 72 h. The coronal brain sections (12 μm) were prepared and mounted onto glass slides for further analysis. After washing with PBS, the sections were incubated at room temperature for 1 h in blocking buffer containing 10% donkey serum and 0.3% Triton X‐100. Subsequently, sections were incubated overnight at 4°C with the following primary antibodies: P2X4R (1:200, 13,534–1‐AP, Proteintech), IBA1 (1:500, DF6442, Affinity), and BDNF (1:500, DF6387, Affinity). The sections were rinsed with PBS the next day and incubated with corresponding secondary antibodies at 37°C for 1 h. The nuclei were counterstained with DAPI (Abcam, ab104135), and the images were captured using a slide scanner.

The immunofluorescence images were analyzed using the FIJI software (NIH). The regions of interest (ROIs) within the target brain areas were randomly selected, and all the images were captured using consistent imaging parameters. The images were converted to grayscale and separated by fluorescence channels. The fluorescence intensity was quantified as the percentage of positive pixels within each ROI. In the colocalization analysis, the IBA1‐positive cells were manually counted in each ROI. The dual‐labeled cells were identified by merging the two individual marker images and counted manually to determine the percentage of colocalization. The microglial morphological analysis was conducted following previously established protocols [30].

### 2.5 Quantitative Real‐Time PCR (qPCR) Analysis

Hippocampal tissues (5–20 mg) were collected from mice and homogenized in precooled tubes containing RNA lysis buffer and grinding beads. The total RNA was extracted using the TRIzol method according to the manufacturer's instructions. Following phase separation with chloroform, isopropanol precipitation, and 75% ethanol wash, the RNA samples were stored at −20°C or used immediately. The RNA concentration and purity were assessed using a NanoDrop 2000 spectrophotometer, with acceptable A260/A280 ratios ranging between 1.8 and 2.0.

Reverse transcription was performed using 200 ng/μL of RNA with the PrimeScript RT reagent kit (Cat. No. G3337, Takara) to synthesize complementary DNA (cDNA). qPCR was conducted using TB Green Premix Ex Taq II (Takara) in a volume of 20 μL following the manufacturer's protocol. Each reaction was performed in technical triplicate. The thermal cycling conditions were as follows: initial denaturation at 95°C for 30 s, followed by 40 cycles of 95°C for 5 s and 60°C for 30 s. A melting curve analysis was performed at the end of the amplification process to confirm specificity.

GAPDH was used as the internal control. The relative expression levels of target genes were calculated using the $2^{-\Delta \Delta C_{t}}$ method and presented as mean ± standard deviation (SD). All the measurements were performed in triplicate. The primer sequences (5′–3′) used for qPCR were as follows:

- M‐GAPDH‐S: CCTCGTCCCGTAGACAAAATG
- M‐GAPDH‐A: TGAGGTCAATGAAGGGGTCGT
- M‐TNFα‐S: CCCTCACACTCACAAACCACC
- M‐TNFα‐A: CTTTGAGATCCATGCCGTTG
- M‐IL6‐S: GACTTCCATCCAGTTGCCTTCT
- M‐IL6‐A: CTCATTTCCACGATTTCCCAGA
- M‐IL1β‐S: AAATGCCACCTTTTGACAGTGA
- M‐IL1β‐A: AAAGAAGGTGCTCATGTCCTCATCC
- M‐P2X4R‐S: GGAAAAGGGCTACCAGGAAAC
- M‐P2X4R‐A: AATGCTGGTCTTATCAGGAATCTCT

### 2.6 Nissl Staining

The frozen brain sections were equilibrated to room temperature for 10 min, followed by immersion in distilled water for 5 min. After gently blotting excess water, the sections were incubated in Solution A of the Nissl Staining Kit (G1086‐100ML, Servicebio) for 15 min. The stained sections were then briefly rinsed in two changes of water (3–5 s). Subsequently, the same kit's sections were differentiated in Solution B (Cresyl Violet Method). Differentiation was monitored under a light microscope and terminated when Nissl bodies were visible and the background was nearly colorless. After lightly blotting, the sections were rapidly dehydrated through three changes of absolute ethanol (3–5 s), cleared in fresh xylene for 2 min, and cover‐slipped with the neutral mounting medium.

### 2.7 Behavioral Assessment

#### 2.7.1 Open Field Test (OFT)

The OFT was conducted to evaluate spontaneous locomotor activity and anxiety‐like behavior in mice. The test was performed in a square open‐field arena (40 cm × 40 cm with 30 cm‐high walls), the floor of which was divided into equal‐sized squares. The central area was defined as the four central squares. Prior to testing, the mice were acclimated to the laboratory environment for at least 30 min. Each mouse was gently placed in the center of the arena, and its behavior was recorded for 5 min using a video tracking system. The total distance traveled was the primary parameter analyzed. After each session, the arena was cleaned with 75% ethanol to eliminate olfactory cues.

#### 2.7.2 Forced Swim Test (FST)

The FST was used to assess the depression‐like behavior in mice. The mice were individually placed in a transparent cylindrical container (20 cm in diameter, 30 cm in height) filled with water to a depth of 15 cm at a temperature of 23°C ± 1°C. Each session lasted 6 min, with the first 2 min considered a habituation period and the last 4 min used for data collection. A video analysis system was used to record the mouse behavior, and the primary outcome was immobility time, which is defined as the duration during which the mouse floated passively with only minimal movements to maintain balance. After the test, the mice were gently dried with a towel and returned to a warming box for recovery.

#### 2.7.3 Tail Suspension Test (TST)

The TST was also employed to assess the depression‐like behavior. The mice were suspended by the tail using adhesive tape placed approximately 1 cm from the tip, with the tail fixed to a suspension bar positioned about 30 cm above the surface. The test lasted for 6 min, with behavior recorded during the last 4 min by a video monitoring system. The immobility time, defined as the total duration during which the mouse ceased struggling and remained completely motionless, was the primary measure. To minimize experimental bias, the testing environment was kept quiet with dim lighting, and no aversive stimuli were applied prior to testing.

### 2.8 Statistical Analysis

All data were collected and analyzed in a blind manner. All data are presented as mean ± standard deviation (SD). Normality and homogeneity of variance were assessed before statistical analysis. For multiple comparisons of more than two groups, data were analyzed using one‐way analysis of variance (ANOVA) followed by Bonferroni's post hoc test with normally distributed or by the Kruskal–Wallis test with non‐normally distributed. Statistics were deemed significant at *p* < 0.05. The body weight data were analyzed by two‐way repeated‐measures ANOVA and Tukey's post hoc test. The Kruskal–Wallis test was used for data that did not meet the assumption of normality or homogeneity of variance, with the post hoc comparison corrected using the Bonferroni method. *p* < 0.05 was considered statistically significant. All the statistical analyses were performed using the GraphPad Prism 10.0 software (GraphPad Prism Software Inc., San Diego, CA, USA).

## 3 Results

### 3.1 LIPUS Alleviation of LPS‐Induced Neuroinflammation in the Hippocampus and Depression‐Like Behavior

**FIGURE 1:**
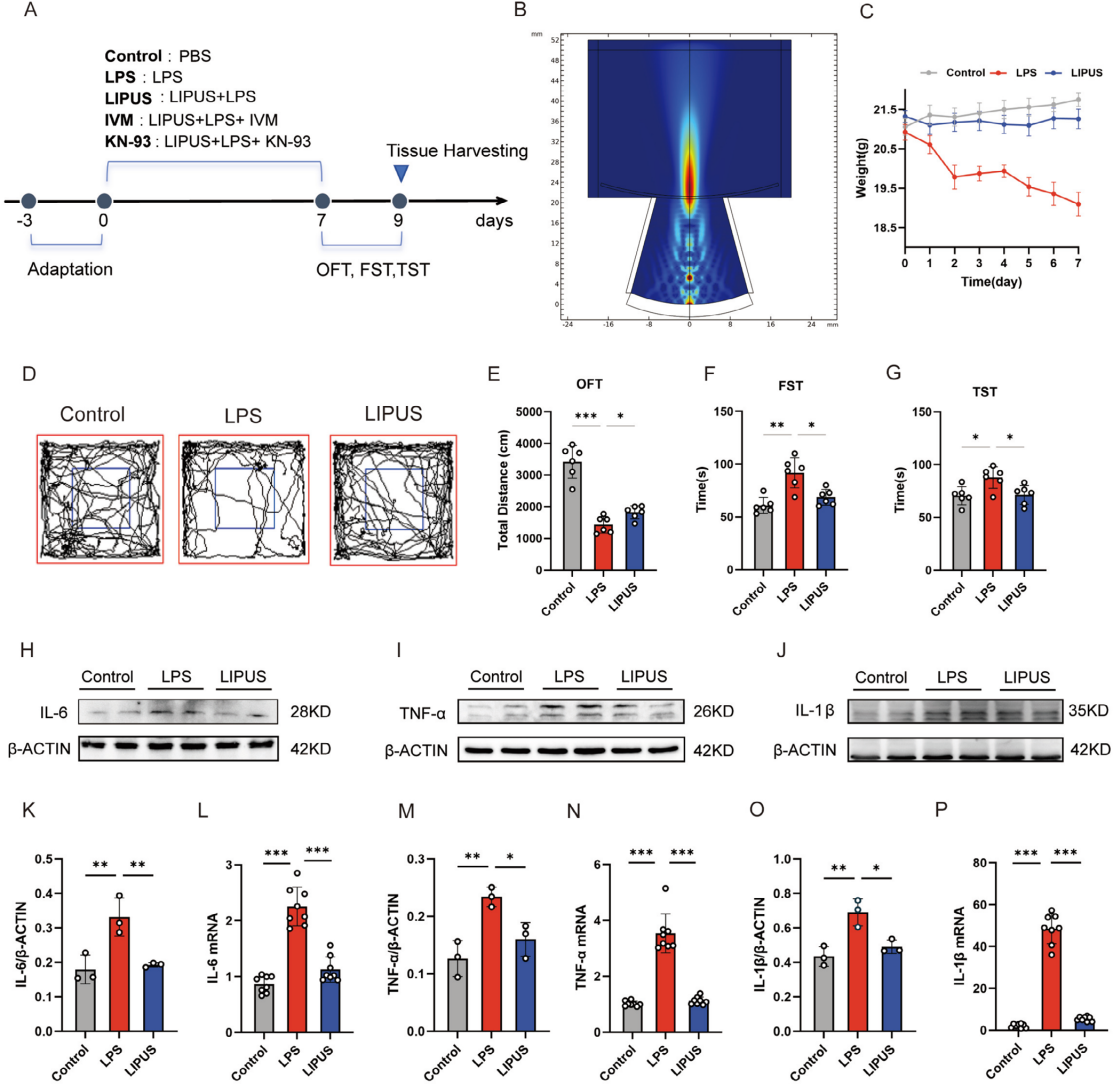
LIPUS attenuation of LPS‐induced hippocampal neuroinflammation and depression‐like behavior: (A) Schematic diagram of the experimental protocol; (B) Ultrasonic probe simulation showing stronger sound intensity at 3 mm beneath the skull, corresponding to the hippocampus; (C) LPS treatment resulting in a downward trend in body weight, which is significantly ameliorated by LIPUS intervention; (D–G) Behavioral assessments, including the OFT, FST, and TST, revealing that LIPUS significantly reverses the LPS‐induced depression‐like behavior; (H–J, K, M, O) Western blot analysis of hippocampal tissue showing that LIPUS suppresses the LPS‐induced upregulation of pro‐inflammatory cytokines IL‐6, IL‐1β, and TNF‐α; (L, N, P) qPCR results further confirming that the mRNA levels of IL‐6, IL‐1β, and TNF‐α followed similar trends as their corresponding protein expressions and supporting the anti‐inflammatory effects of LIPUS. LPS, Lipopolysaccharide; LIPUS, Low‐intensity pulsed ultrasound; IVM, Ivermectin; KN‐93, CaMKII antagonist; OFT, Open field test; FST, Forced swim test; TST, Tail suspension test. The *p* value was determined by ANOVA with Bonferroni's post‐hoc test. *p < 0.05, **p < 0.01, ***p < 0.001. Data are represented as mean ± SD.

To investigate the therapeutic effects of LIPUS on LPS‐induced neuroinflammation and depression‐like behavior, a mouse model of depression is established by systemic LPS administration, followed by targeted LIPUS intervention at defined time points (Figure 1A). The ultrasonic probe simulation demonstrates the sound intensity beneath the mouse skull, revealing a stronger intensity at a depth of 3 mm, which corresponds to the depth of the hippocampus in mice (Figure 1B). LPS treatment results in a downward trend in body weight, whereas mice in the LIPUS group maintain their normal weight (Figure 1C). Behavioral assessment reveals that, compared with the control group, LPS‐treated mice exhibit a significant increase in locomotor activity in the OFT, as well as prolonged immobility times in both the forced swim test (FST) and tail suspension test (TST), indicating robust depression‐like behavior. Notably, the LIPUS treatment markedly reverses these behavioral alterations, and the mice in the LIPUS group deliver improved performance across all three behavioral paradigms compared with the LPS group (Figure 1D–G).

At the molecular level, the expression of pro‐inflammatory cytokines IL‐6, IL‐1β, and TNF‐α in hippocampal tissue is determined to evaluate the degree of neuroinflammation. Western blot analysis shows that LPS significantly increases the protein levels of these cytokines, while LIPUS treatment effectively suppresses their upregulation (Figure 1H–J,K,M,O). Consistently, qPCR results confirm that the mRNA expression patterns of IL‐6, IL‐1β, and TNF‐α mirrored their protein expression trends (Figure 1L,N,P), indicating that LIPUS effectively attenuates LPS‐induced hippocampal inflammation.

### 3.2 LIPUS Suppressing LPS‐Induced Neuroinflammation by Regulating P2X4R Expression in Microglia

**FIGURE 2:**
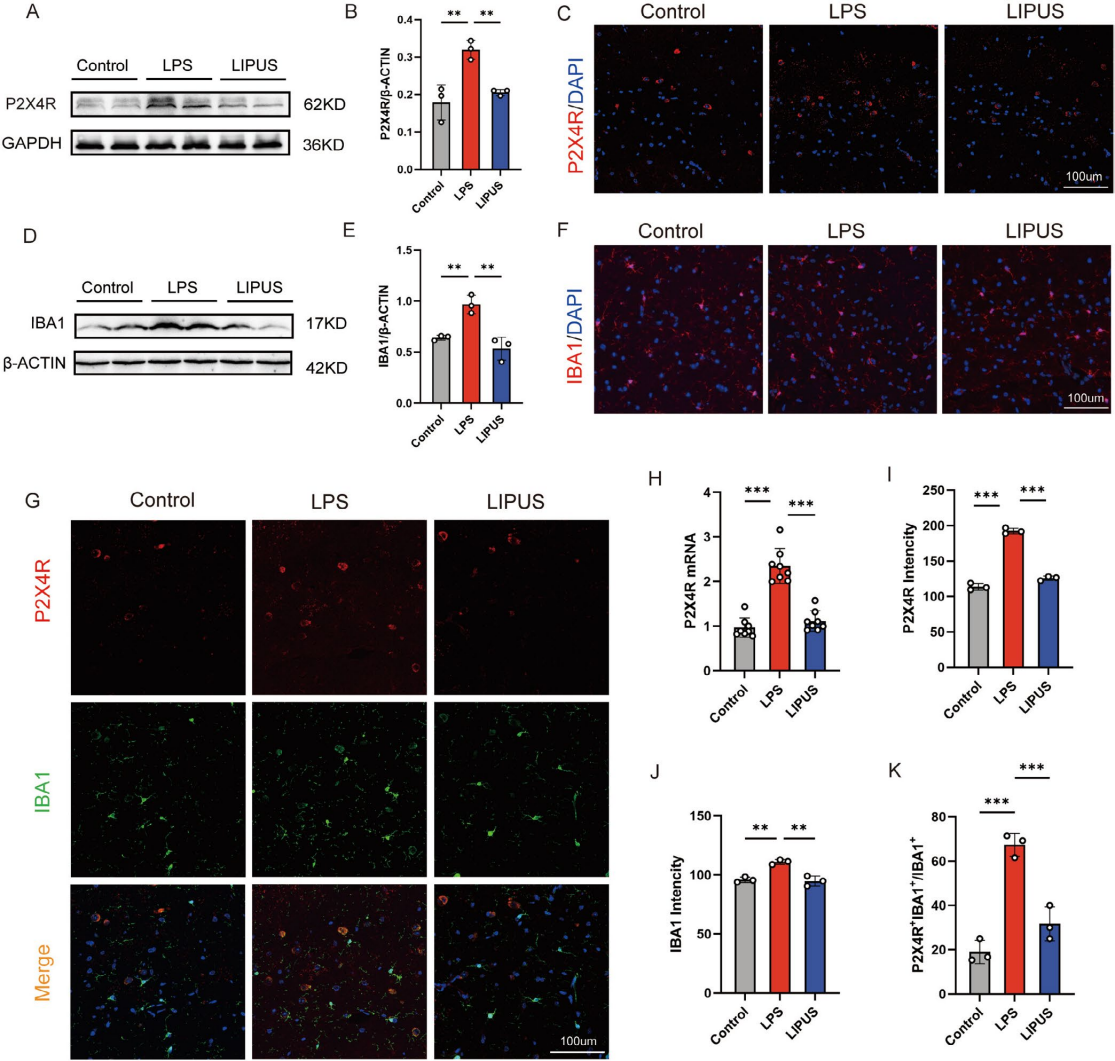
LIPUS suppressing LPS‐induced neuroinflammation by modulating P2X4R expression in microglia: (A, B) Western blot analysis showing that LPS significantly increased P2X4R protein expression in the hippocampus, which is markedly inhibited by LIPUS treatment; (C, I) Immunofluorescence staining confirming elevated hippocampal P2X4R expression following LPS administration, which is reduced after LIPUS intervention; (D, E) Western blot analysis showing that LPS markedly upregulates the microglial marker IBA1, while LIPUS significantly suppresses the upregulation; (F, J) Immunofluorescence results validating the reduction in IBA1 expression following LIPUS treatment, indicating decreased microglial activation; (G, K) Dual immunofluorescence staining for P2X4R and IBA1 revealing strong colocalization in the LPS group, suggesting that P2X4R is primarily expressed in activated microglia and the LIPUS treatment markedly reduces colocalization, supporting that LIPUS mitigates neuroinflammation by inhibiting P2X4R activation in microglia; (H) PCR analysis showing that P2X4R expression is significantly elevated following LPS administration, which is reduced after LIPUS intervention. LPS, Lipopolysaccharide; LIPUS, Low‐intensity pulsed ultrasound. The *p* value was determined by ANOVA with Kruskal–Wallis test. *p < 0.05, **p < 0.01, ***p < 0.001. Data are represented as mean ± SD.

To investigate the role of LIPUS in modulating P2X4R channel activity and its cellular localization, the changes in P2X4R expression in the hippocampus are assessed. Western blot analysis reveals that LPS significantly upregulates the P2X4R protein expression, while this treatment markedly attenuates the LIPUS effect (Figure 2A,B). Meanwhile, qPCR results further demonstrate that LPS also significantly increased P2X4R mRNA levels, whereas LIPUS treatment markedly decreased/partially restored its expression (Figure 2H). Immunofluorescence staining further confirms an elevated P2X4R expression in the LPS group, which is substantially reduced following LIPUS intervention (Figure 2C,I).

Owing to prior evidence that P2X4R is predominantly expressed in microglia, the expression of IBA1, a well‐established microglial marker, is examined. Western blot and immunofluorescence results indicate that LPS significantly increases IBA1 expression, whereas LIPUS suppresses the upregulation (Figure 2D–F,J), suggesting that LIPUS modulates microglial activation. To further validate the microglial localization of P2X4R, dual immunofluorescence staining is performed for P2X4R and IBA1. The results demonstrate strong colocalization of P2X4R with IBA1 in the hippocampus of LPS‐treated mice, indicating that P2X4R is mainly expressed in activated microglia. Notably, LIPUS significantly reduces the extent of colocalization (Figure 2G,K). The findings support how LIPUS attenuates neuroinflammation by suppressing P2X4R activity in microglia.

### 3.3 LIPUS Inhibition of Neuronal Apoptosis via Activation of the BDNF/TrkB Signaling Pathway

**FIGURE 3:**
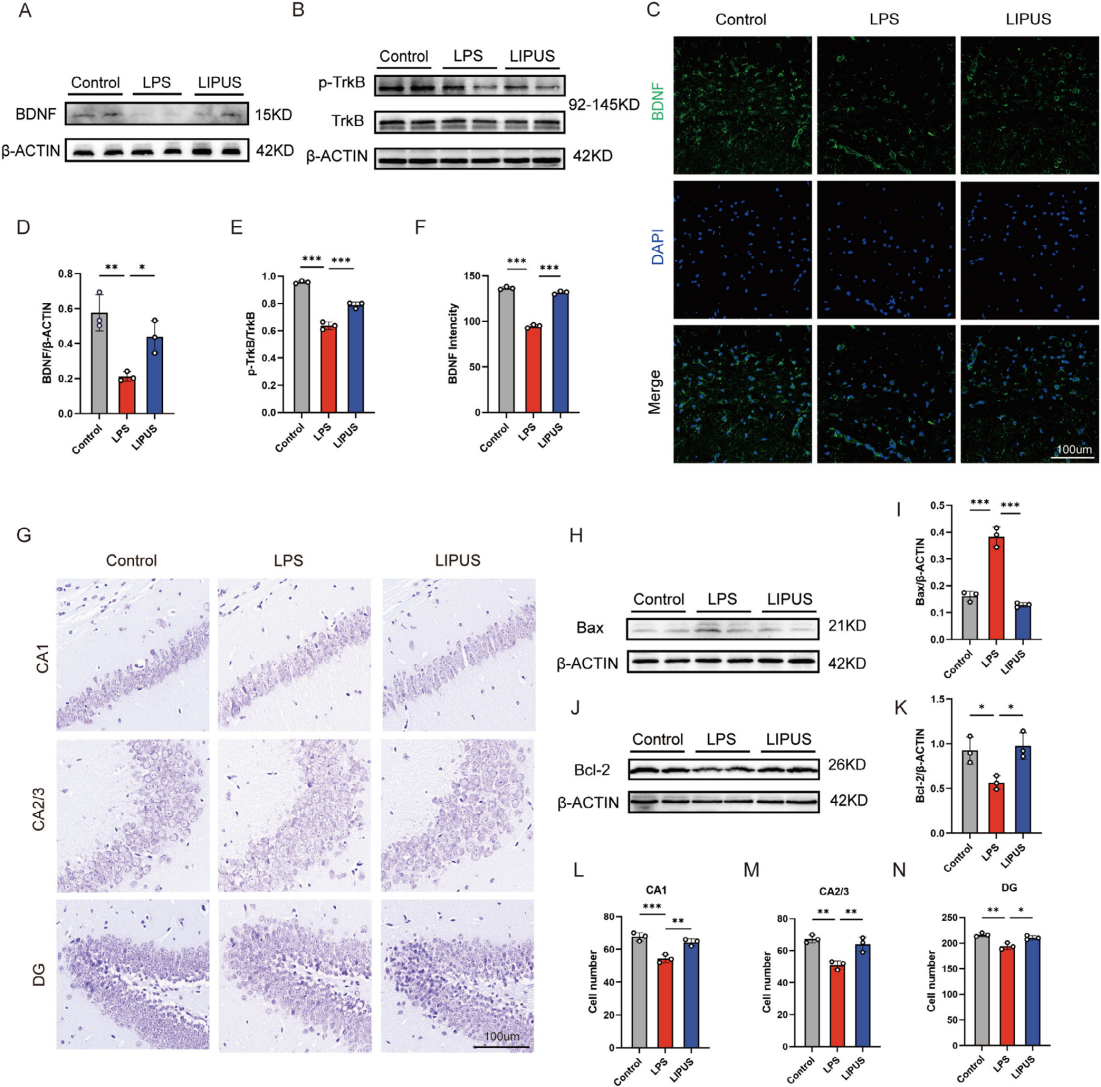
LIPUS inhibition of LPS‐induced neuronal apoptosis by activating the BDNF/TrkB signaling pathway: (A, D) Western blot results showing that LPS significantly downregulates BDNF protein expression in the hippocampus, whereas LIPUS treatment markedly increases the BDNF levels; (B, E) Total TrkB protein levels not significantly different among groups but LIPUS increasing the expression of p‐TrkB; (C, F) Immunofluorescence staining confirming the enhancement of hippocampal BDNF expression following LIPUS intervention; (G, L–N) Nissl staining revealing disorganized neuronal arrangement and reduced cell numbers in the CA1, CA2/3, and DG hippocampus regions in the LPS group, showing that these structural impairments are substantially improved after LIPUS treatment; (H–K) Western blot analysis showing that LPS increases the expression of the pro‐apoptotic protein Bax and decreases the expression of the anti‐apoptotic protein Bcl‐2 and LIPUS significantly reversing these effects, indicating its anti‐apoptotic potential. LPS, Lipopolysaccharide; LIPUS, Low‐intensity pulsed ultrasound. The *p* value was determined by ANOVA with Bonferroni's post‐hoc test. *p < 0.05, **p < 0.01, ***p < 0.001. Data are represented as mean ± SD.

To explore the potential molecular mechanisms of the antidepressant effects of LIPUS, the expression levels of BDNF and its receptor TrkB are determined. Western blotting reveals that LPS administration significantly downregulates the BDNF protein expression in the hippocampus, whereas the LIPUS treatment reverses this suppression and markedly elevates the BDNF levels (Figure 3A,D). Immunofluorescence staining confirms a significantly reduced BDNF expression in the LPS group, with a notable increase in BDNF fluorescence intensity following the LIPUS treatment (Figure 3C,F), suggesting that LIPUS promotes BDNF expression.

The TrkB receptor expression and its phosphorylation status are further evaluated. While the total TrkB protein levels do not differ significantly among groups, the phosphorylated TrkB (p‐TrkB) levels decrease considerably in the LPS group and increase substantially in the LIPUS group (Figure 3B,E), indicating that LIPUS activates the TrkB signaling pathway.

Since the BDNF/TrkB pathway plays a critical role in neuronal survival and anti‐apoptotic regulation, especially in CNS injury and depression‐related models, its involvement in the neuroprotective effects of LIPUS is studied. Western blotting of the apoptosis‐related markers shows that LPS upregulates the pro‐apoptotic protein Bax and downregulates the anti‐apoptotic protein Bcl‐2, suggesting increased hippocampal neuronal apoptosis. In contrast, LIPUS treatment suppresses the Bax expression and enhances the Bcl‐2 expression, indicating a strong anti‐apoptotic effect (Figure 3H–K). Nissl staining further demonstrates that the LPS‐treated mice exhibit disorganized neuronal arrangement, reduced cell number, and weaker staining intensity in the CA1, CA2/3, and DG regions of the hippocampus. LIPUS ameliorates these structural damages, with improved neuronal organization and morphology (Figure 3G,L–N). Together, these results suggest that LIPUS produces neuroprotective effects by enhancing BDNF/TrkB signaling and inhibiting neuronal apoptosis.

### 3.4 P2X4R Agonist Attenuating the Enhancing Effect of LIPUS on the BDNF/TrkB Signaling Pathway

**FIGURE 4:**
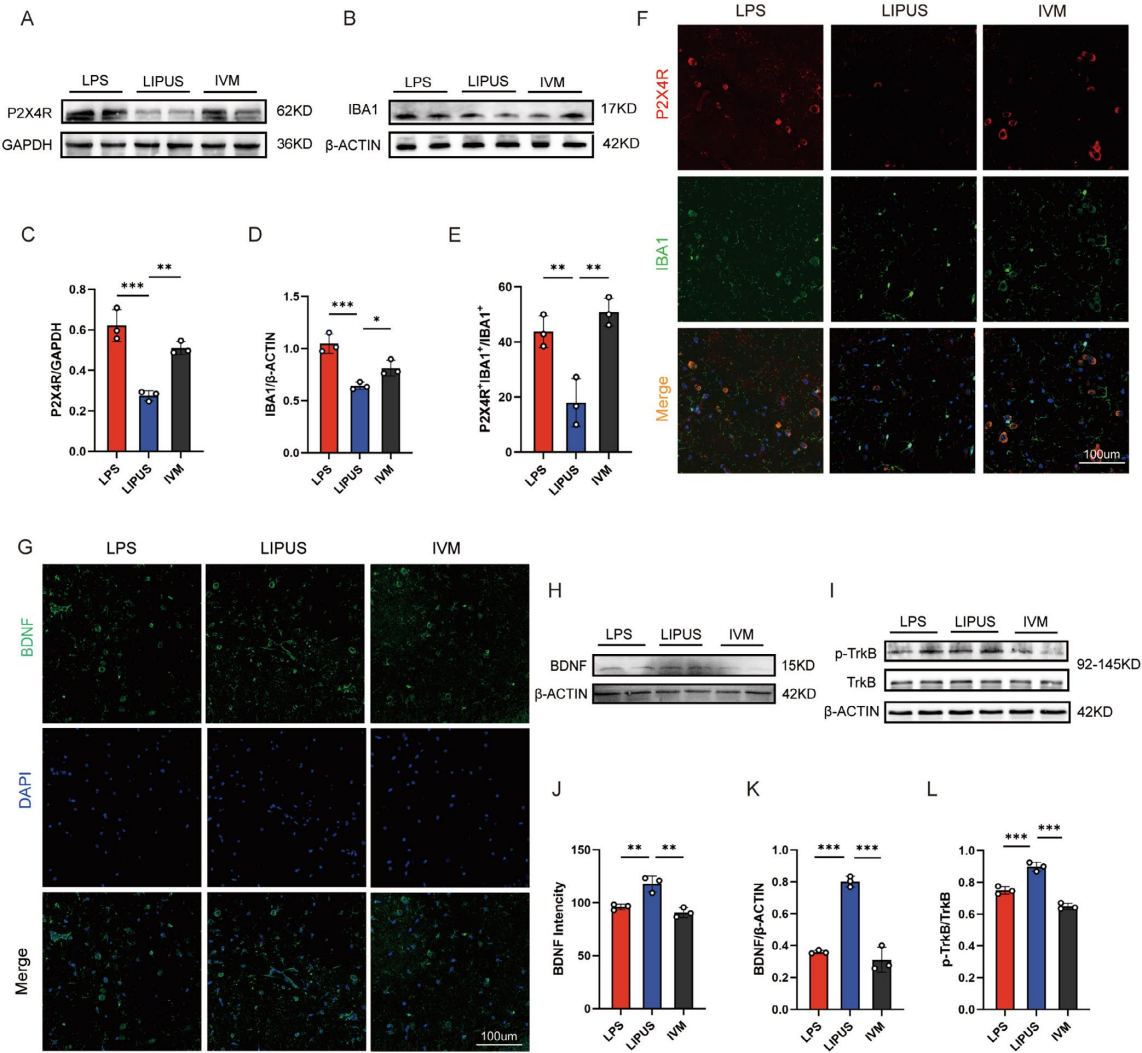
P2X4R agonist attenuating the LIPUS‐induced enhancement of BDNF/TrkB signaling and neuroprotection: (A–C) Western blot analysis showing that LIPUS significantly suppresses the LPS‐induced P2X4R expression, while co‐administration of the P2X4R agonist ivermectin (IVM) reverses the inhibitory effect; (B–D) Western blot analysis of IBA1 revealing that LIPUS markedly reduces LPS‐induced microglial activation, whereas IVM restores the IBA1 expression levels; (E, F) Dual immunofluorescence staining for P2X4R and IBA1 demonstrating that colocalization decreases in the LIPUS group but increases in the IVM group, indicating that IVM restores both the P2X4R expression and microglial activation; (G, J) Immunofluorescence staining showing that LIPUS significantly enhances BDNF expression in the hippocampus, whereas this effect is markedly reduced following IVM administration; (H, K) Western blot analysis revealing that LIPUS upregulates the BDNF protein levels, while the BDNF expression decreases in the IVM group; (I, L) Despite unchanged total TrkB levels in the three groups, LIPUS increases p‐TrkB with IVM suppressing. LPS, Lipopolysaccharide; LIPUS, Low‐intensity pulsed ultrasound; IVM, Ivermectin. The *p* value was determined by ANOVA with Bonferroni's post‐hoc test. *p < 0.05, **p < 0.01, ***p < 0.001. Data are represented as mean ± SD.

To further verify the anti‐inflammatory mechanism of LIPUS via modulation of the P2X4R activity, a selective P2X4R agonist, IVM, is co‐administered during the LIPUS treatment. The mice are divided into three groups: LPS, LIPUS, and IVM, and the molecular and cellular experiments described earlier are repeated. Western blot analysis reveals that the P2X4R protein expression is significantly lower in the LIPUS group compared to the LPS group. However, in the IVM group, the P2X4R expression increases again to levels comparable to that of the LPS group (Figure 4A–C), indicating that IVM can reverse the LIPUS‐induced suppression of P2X4R. Regarding microglial activation, Western blotting shows that LIPUS significantly reduces the LPS‐induced expression of the microglial marker IBA1, while the IVM treatment restores the IBA1 levels (Figure 4B–D), suggesting that P2X4R activation blocks the inhibitory effect of LIPUS on microglial activation. Furthermore, dual immunofluorescence staining reveals a marked reduction in the colocalization of P2X4R and IBA1 in the LIPUS group, while the IVM group shows a significant restoration of this colocalization signal (Figure 4E,F). This indicates that P2X4R activation restores microglial activity and P2X4R expression induced by LPS.

Western blot analysis shows that LPS significantly suppresses the BDNF expression, whereas the LIPUS treatment markedly upregulates the BDNF protein levels. However, the BDNF expression decreases significantly in the IVM group compared to the LIPUS group (Figure 4H,K). Immunofluorescence staining further confirms that LIPUS enhances the hippocampal BDNF signals, while this effect diminishes following IVM administration (Figure 4G,J). With regard to TrkB signaling, no significant difference is observed in the total TrkB protein levels among the three groups. However, LIPUS increases the p‐TrkB expression, which is reversed by IVM co‐treatment, resulting in reduced p‐TrkB levels compared to the LIPUS group (Figure 4I,L).

### 3.5 P2X4R Regulates the BDNF/TrkB Signaling Pathway and Apoptosis by Modulating CaMKII/CREB Phosphorylation in the Hippocampus

**FIGURE 5:**
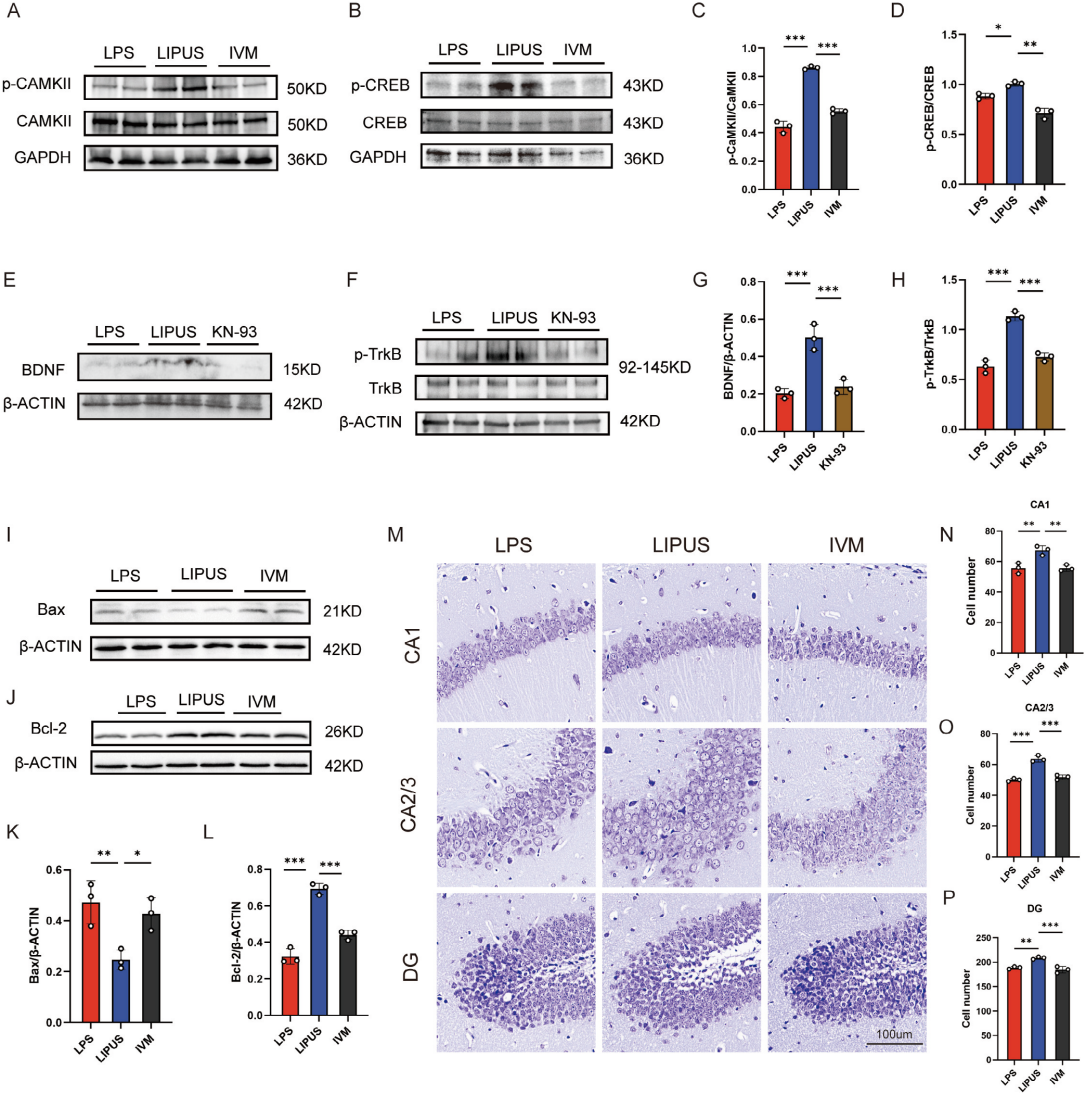
P2X4R regulates the BDNF/TrkB signaling pathway and apoptosis by modulating CaMKII/CREB phosphorylation in the hippocampus: (A, C) Western blot analysis revealing that LIPUS modulates CaMKII phosphorylation, as indicated by the p‐CaMKII/CaMKII ratio, while CaMKII phosphorylation is decreased in the IVM group; (B, D) Western blot analysis revealing that LIPUS modulates CREB phosphorylation, as indicated by the p‐CREB/CREB ratio, while CREB phosphorylation is decreased in the IVM group; (E, G) Western blot analysis revealing that KN‐93 administration (CaMKII antagonist) markedly reduces BDNF protein expression, as shown by the representative immunoblots and densitometric quantification; (F, H) Western blot analysis revealing that KN‐93 administration markedly decreases TrkB phosphorylation, as indicated by the p‐TrkB/TrkB ratio, together with the representative immunoblots and quantitative analysis; (I–L) Western blot results indicating that LIPUS downregulates the pro‐apoptotic protein Bax and upregulates the anti‐apoptotic protein Bcl‐2, demonstrating a neuroprotective effect. This trend is reversed by IVM, as shown by increased Bax and decreased Bcl‐2 expression; (M–P) Nissl staining showing that LIPUS improves the hippocampal neuronal structure and co‐treatment with IVM resulting in disorganized neuronal arrangement and morphological degeneration, resembling that of the LPS group. LPS, Lipopolysaccharide; LIPUS, Low‐intensity pulsed ultrasound; IVM, Ivermectin; KN‐93: CaMKII antagonist. The *p* value was determined by ANOVA with Bonferroni's post‐hoc test. *p < 0.05, **p < 0.01, ***p < 0.001. Data are represented as mean ± SD.

To further elucidate whether P2X4R influences the BDNF/TrkB signaling pathway and apoptosis by modulating CaMKII/CREB phosphorylation, we examined the phosphorylation status of CaMKII and CREB in the hippocampus and the alterations of downstream molecules. Western blot analysis showed that LIPUS markedly modulated CaMKII phosphorylation, whereas CaMKII phosphorylation was significantly reduced following IVM administration (Figure 5A,C). Consistently, LIPUS also modulated CREB phosphorylation; however, IVM treatment significantly decreased CREB phosphorylation, suggesting that P2X4R activation suppresses phosphorylation of the CaMKII/CREB axis (Figure 5B,D). To further verify the critical role of CaMKII in this pathway, the CaMKII antagonist KN‐93 was administered. Notably, KN‐93 markedly reduced BDNF protein expression (Figure 5E,G) and significantly inhibited TrkB phosphorylation (Figure 5F,H), indicating that CaMKII activity is essential for maintaining/promoting BDNF expression and TrkB phosphorylation.

Additionally, analysis of apoptosis‐related proteins reveals that LIPUS significantly downregulates the pro‐apoptotic protein Bax and upregulates the anti‐apoptotic protein Bcl‐2, indicating a neuroprotective trend. In contrast, the IVM treatment increases Bax and decreases Bcl‐2 expression levels, resembling the profile observed from the LPS group (Figure 5I–L). Nissl staining further supports these findings, showing that LIPUS improves neuronal structural integrity, while the IVM group exhibits disrupted neuronal arrangement and degenerative morphology similar to that of the LPS group (Figure 5M–P).

These results suggest that activation of P2X4R weakens the neuroprotective effects of LIPUS by downregulating the BDNF–TrkB signaling pathway and promoting neuronal apoptosis, underscoring the critical negative regulatory role of P2X4R in LIPUS‐mediated antidepressant and neuroprotective mechanisms.

### 3.6 P2X4R Agonist Attenuating the Antidepressant and Anti‐Inflammatory Effects of LIPUS

**FIGURE 6:**
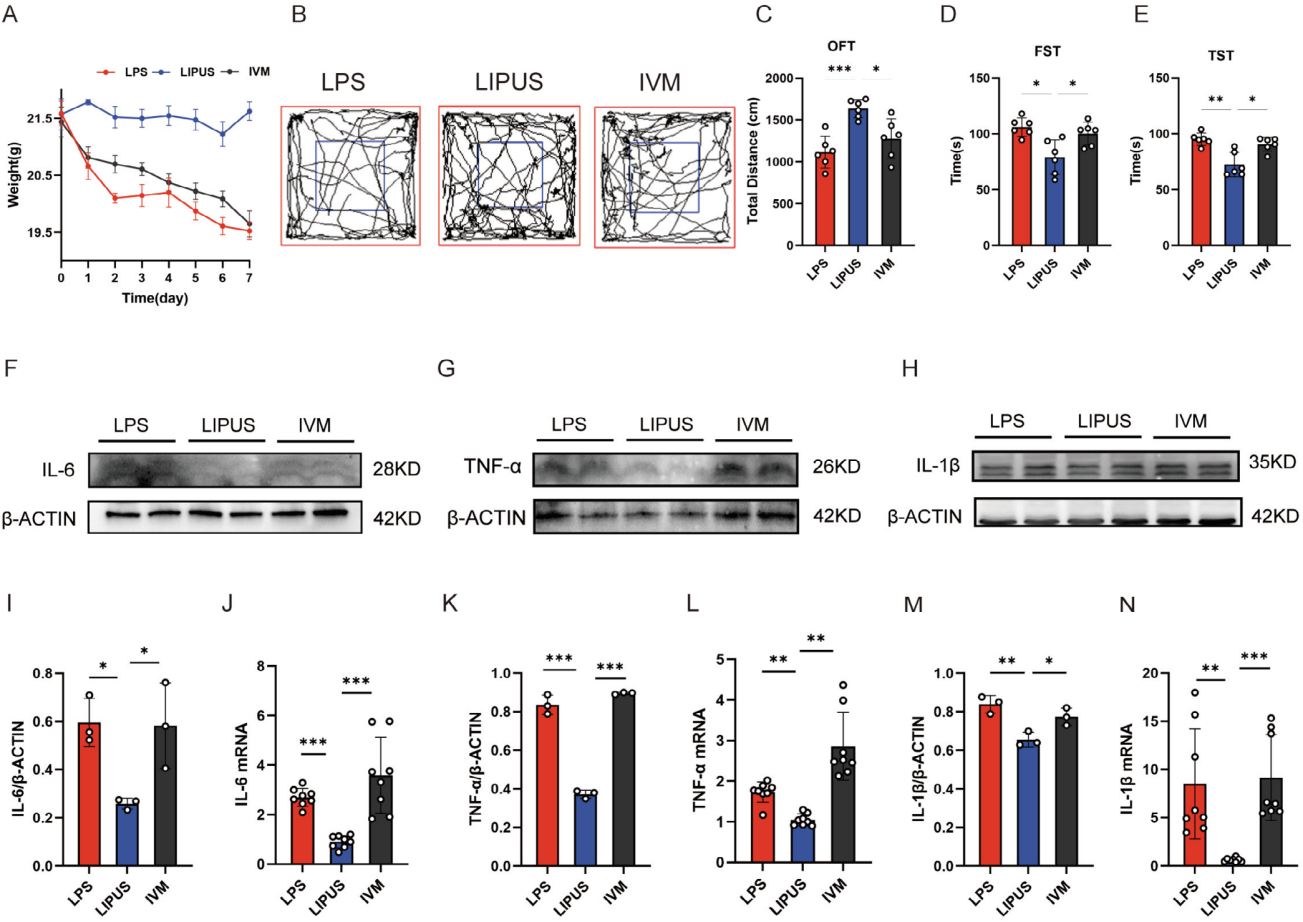
P2X4R agonist IVM attenuating the beneficial effects of LIPUS on depression‐like behavior and neuroinflammation: (A) LIPUS significantly improving the LPS‐induced body weight loss in mice and co‐administration of IVM reversing this improvement; (B–E) Behavioral assessment showing that LIPUS reduces the depression‐like behavior, as evidenced by decreased immobility time and increased time spent in the central zone. In contrast, the IVM group exhibits behavioral patterns similar to the LPS group, indicating that IVM weakens the antidepressant effects of LIPUS; (F–H, I, K, M) Western blot analysis revealing that LIPUS suppressing the expressions of IL‐1β, IL‐6, and TNF‐α with co‐treatment with IVM leading to a notable rebound in these pro‐inflammatory cytokines. (J, L, N) qPCR analysis further confirming that IVM attenuates the LIPUS‐mediated suppression of IL‐1β, IL‐6, and TNF‐α mRNA expression. LPS, Lipopolysaccharide; LIPUS, Low‐intensity pulsed ultrasound; IVM, Ivermectin. The *p* value was determined by ANOVA with Bonferroni's post‐hoc test. *p < 0.05, **p < 0.01, ***p < 0.001. Data are represented as mean ± SD.

Administration of IVM reverses the LIPUS‐induced improvement in the body weight of the LPS‐treated mice (Figure 6A). Behavioral assessment shows that LIPUS alleviates the depression‐like behavior compared to the LPS group. However, in the IVM group, the improvement diminishes. The increased immobility time and reduced time spent in the central zone approach those of the LPS group. Statistical analysis indicates that IVM partially reverses the antidepressant effects of LIPUS (Figure 6B–E).

The changes in the inflammatory cytokine expression further corroborate these findings. Western blot and qPCR analyses demonstrated that LIPUS suppresses the LPS‐induced IL‐1β, IL‐6, and TNF‐α expression. However, in the IVM group, the expression levels of these cytokines are elevated again with statistically significant differences (Figure 6F–N), suggesting that activation of P2X4R weakens the inhibitory effect of LIPUS on hippocampal neuroinflammation.

## 4 Discussion

In this study, the behavioral effects and potential neuroimmune mechanisms of LIPUS in a mouse model of LPS‐induced depression are studied systematically. The results demonstrate that LIPUS significantly alleviates the depression‐like behavior and reduces the pro‐inflammatory cytokine expression in the hippocampus. Mechanistically, LIPUS produces neuroprotective and neuromodulatory effects by regulating the microglial P2X4R activity and enhancing the BDNF/TrkB signaling pathway.

Depression is a complex psychiatric disorder characterized by persistent low mood, cognitive dysfunction, and behavioral alterations. However, its underlying pathophysiological mechanisms are still not well understood. In recent years, the neuroinflammation hypothesis has garnered increasing attention. A growing body of evidence indicates that levels of pro‐inflammatory cytokines, such as IL‐1β, IL‐6, and TNF‐α, are elevated in both the peripheral and central nervous systems of individuals with depression [10], suggesting that inflammatory responses contribute to the onset and progression of the disorder. During this process, the hippocampus plays a crucial role in emotion regulation and cognitive function, and its structural and functional abnormalities are considered a core component in the development of depression [31, 32]. Studies have shown that inflammatory cytokines can disrupt hippocampal neurogenesis [33], suppress BDNF expression [34], and promote neuronal apoptosis, leading to hippocampal atrophy and functional circuit impairment. The BDNF/TrkB signaling pathway, a key molecular mechanism that governs synaptic plasticity, neurogenesis, and neuronal survival [35, 36], plays a pivotal role in the pathophysiology of depression. Inflammatory factors can attenuate the neuroprotective functions of this pathway by downregulating BDNF and its receptor TrkB and reducing TrkB phosphorylation [37] to exacerbate hippocampal damage. Suppression of the BDNF/TrkB pathway not only diminishes neuronal viability [38] and synaptic plasticity [39] but also impairs emotional regulation and stress responsiveness, contributing to the emergence of depression‐like behavior. Moreover, inflammatory signaling may further aggravate these symptoms by activating microglia [40], promoting cellular apoptosis [41], and disrupting the neural network integrity.

LIPUS, a non‐invasive physical modality that produces no thermal effects, has recently shown promise in neurological disorders. Previous studies have indicated that LIPUS can penetrate the skull and modulate neural excitability [42, 43], cerebral perfusion, and intracellular signaling [44, 45] to influence mood and memory. In this study, LIPUS is found to alleviate the LPS‐induced depression‐like behavior in mice, likely by the modulation of inflammation [46], activation of the BDNF/TrkB pathway [47], and enhancement of synaptic plasticity. Our findings suggest that LIPUS may attenuate neuroinflammation by modulating glial cell function, consequently restoring the balance between inflammation and neural circuits, a concept that provides both a mechanistic foundation and a therapeutic rationale for its application in depression.

It is further demonstrated that LIPUS strongly downregulates the hippocampal TNF‐α, IL‐1β, and IL‐6 expression and suppresses the microglial activation in the LPS model [48, 49]. Concurrently, BDNF expression is upregulated, consistent with previous findings [47], and likely contributes to the observed neuroprotection. Restoring neurotrophic support may facilitate neural repair and functional recovery to ultimately improve depression phenotypes. The results show that LIPUS not only improves the behavioral deficits but also produces substantial anti‐inflammatory effects on the molecular level, further supporting the pivotal role of inflammation in depression. P2X4R, an ATP‐gated ion channel predominantly expressed in microglia [50, 51], has emerged as a critical link between neuroinflammation and depression [20]. It has been implicated in neuroimmune modulation in disorders such as Parkinson's disease [45].

Notably, increasing evidence over recent years has begun to elucidate how P2X4R may mechanistically interface with the BDNF/TrkB pathway. As a Ca^2+^‐permeable, ATP‐gated ion channel, activation of P2X4R can evoke intracellular Ca^2+^ signaling dynamics and subsequently modulate CaMKII activity and downstream CREB phosphorylation, thereby shaping inflammation‐related transcriptional programs and cellular functional states. Importantly, this regulation appears to be context‐dependent and potentially bidirectional, such that P2X4R activation may either enhance or suppress CaMKII/CREB phosphorylation depending on the cellular milieu and inflammatory tone [52, 53]. In parallel, converging studies have established the CaMKII/CREB phosphorylation axis as a core transcriptional regulatory module governing neurotrophin expression; in particular, p‐CREB–driven activity‐dependent transcription directly promotes BDNF expression, which in turn facilitates TrkB receptor activation and downstream neurotrophic signaling [54, 55]. Moreover, under chronic inflammatory stimulation, microglial P2X4R has been reported to regulate BDNF release via TrkB‐dependent mechanisms [19]. In the present study, our data further substantiate the coherence of this signaling cascade in an inflammation‐associated depression context: following LPS challenge, aberrant P2X4R activation was accompanied by suppressed CaMKII/CREB phosphorylation and concomitant attenuation of BDNF/TrkB signaling. Conversely, LIPUS suppressed pathological P2X4R activation, normalized CaMKII/CREB phosphorylation status, and synchronously restored BDNF expression and TrkB activation, thereby coupling anti‐inflammatory actions with behavioral improvement.

It is noted that despite the comprehensive findings, the depression model is designed via LPS to mimic neuroinflammation and may differ from chronic stress–related depression pathologies in clinical settings. Future work using chronic unpredictable mild stress (CUMS) and other classical depression models may be needed to enhance translational relevance. Moreover, although P2X4R is identified to be a key node, the precise upstream and downstream signaling pathways require further elaboration. Nonetheless, in conclusion, this study reveals that LIPUS ameliorates the depression‐like behavior by suppressing P2X4R activation in microglia, reducing neuroinflammation, and enhancing the BDNF/TrkB neurotrophic pathway. The new knowledge provides compelling evidence for the utility of LIPUS as a non‐invasive neuromodulatory strategy and establishes P2X4R as a potential molecular target for multidimensional, precision‐guided antidepressant therapies.

## Funding

This research was supported by the Guangdong Basic and Applied Basic Research Foundation, China (2021A1515010135), the IIT Research Project of The Fifth Affiliated Hospital of Sun Yat‐sen University (YNZZ 2021‐09), the 2024 Young Backbone Teachers Overseas Study and Training Funding Project (JWFX2024004), the Anhui Province Science and Technology Innovation Challenge Plan (202423l10050047), the 2022 Natural Science Foundation of Anhui Province (2208085MH254), the College Student Innovation and Entrepreneurship Program (202310366032), and the China Scholarship Council (202406380308). Thanks to the Department of Rehabilitation Medicine at the Fifth Affiliated Hospital of Sun Yat‐sen University for their funding support of this study as a flagship department for Chinese and Western medicine collaboration.

## Ethics Statement

The protocols were reviewed and approved by the Ethics Committee of Anhui Medical University (Approval No. LLSC20232258).

## Conflicts of Interest

The authors declare no conflicts of interest.

## Data Availability

The data that support the findings of this study are available from the corresponding author upon reasonable request.
